# Development of focal segmental glomerulosclerosis in a patient with Familial Mediterranean Fever resistant to colchicine therapy under treatment with Canakinumab

**DOI:** 10.1186/1546-0096-13-S1-P95

**Published:** 2015-09-28

**Authors:** K Barut, N Canpolat, A Adrovic, R Cicek, AB Sinoplu, E Arslan, O Kasapcopur

**Affiliations:** 1 Istanbul University, Cerrahpasa Medical Faculty, Pediatric Rheumatology, Istanbul, Turkey; 2 Istanbul University, Cerrahpasa Medical Faculty, Pediatric Nephrology, Istanbul, Turkey

## Introduction

Familial Mediterranean Fever (FMF) represents an autoinflammatory disease caused be MEFV gene mutation characterized with attacks of polyserositis, commonly seen among Turks, Arabs, Armenians and Jews. Therapy with colchicine was proven to be effective in treatment of FMF polyserositis and in prevention of amyloidosis development. Colchicine resistant FMF is defined as 6 or more attacks of polyserositis in a year, despite the regular and sufficient usage of colchicine. The percentage of resistant FMF has been reported as a 5-10% in the literature. Although colchicine remains the gold standard for the FMF treatment, anti-IL-1 drugs are used in the cases of FMF resistant to colchicine therapy. Particularly carriers of the M694V homozygous mutation prone to be resistant to standard therapy and show a risk for development of additional diseases.

## Objective

In this case report, we are presenting a M694V homozygous, colchicine resistant FMF patients with the diagnosis of inflammatory bowel disease, who developed proteinuria, and who was consequently diagnosed with focal segmental glomerulosclerosis (FSGS).

## Case report

A two years old female patient, admitted to our hospital with high fever and severe abdominal pain. She was complaining of high fever (until 39^0^ C) lasting for 3 days accompanied with severe abdominal pain, being repeated every week for the last 6 months. The patient was hospitalized at Gastroenterology department where she was diagnosed as an inflammatory bowel disease and treated with sulphasalazine and low dose prednisone. No clinical response to the therapy was a reason for rheumatologic consultation. Clinical findings of the patient and her higher acute phase reactant levels are compatible with FMF. Colchicine 1 mg/day was started with 1 mg/day. MEFV gene analysis was revealed homozygote M694V mutations. In spite of initial clinical improvement, attacks of disease started to repeat so the patient was consider to be a resistant to standard therapy so the therapy with Anakinra 50mg/day was initiated. Because of partial clinical response to Anakinra, canakinumab 5 mg/kg/dose once in every two months was added. As a result, no disease attack was registered during the time period of one with decreased acute phase reactants to normal value. In further follow up of patient, disease attacks restarted so the canakinumab dose interval was decreased to be given once per month. Although the initial analysis of urine was found to be normal, control urine tests revealed a proteinuria and hematuria. The renal ultrasound examination was normal. Serological tests: ANA, Anti-ds-DNA, C3, C4 levels were normal. Daily protein excretion was measured as a 625 mg/day. Persisting proteinuria and hematuria were a reason to perform a renal biopsy's which suspected amyloidosis. Pathological examination showed mesengial cell proliferation and segmental sclerosis by PAS stain. Congo stain was negative for amyloidosis. Proteinuria together with mentioned pathological findings was compatible for FSGS. Since the pulse prednisone therapy showed no clinical improvement, cyclosporine A was added in therapy. FMF attacks (fever, abdominal pain) restarted so the canakinumab dose was increased to a 65 mg/day. Attacks were disappeared.

## Conclusion

Amyloidosis and other renal pathological conditions should be suspected in the case of proteinuria developed at FMF patient being treated regularly with colchicine and other treatment modalities. It is important to consider a FSGS in differential diagnosis of FMF patients with renal involvement.

## Consent to publish

Written informated consent for publication of their clinical details was obtained from the patient/parent/guardian/relative of the patient.

